# Changes in complement alternative pathway components, factor B and factor H during dengue virus infection in the AG129 mouse

**DOI:** 10.1099/jgv.0.001547

**Published:** 2021-01-07

**Authors:** Sheila Cabezas-Falcon, Aidan J. Norbury, Jarrod Hulme-Jones, Sonja Klebe, Penelope Adamson, Penny A. Rudd, Suresh Mahalingam, Li-Ching Ong, Sylvie Alonso, David L. Gordon, Jillian M. Carr

**Affiliations:** ^1^​ Microbiology and Infectious Diseases, Flinders University, Bedford Park, Adelaide 5042, South Australia; ^2^​ Anatomy and Pathology, College of Medicine and Public Health, Flinders University, Bedford Park, Adelaide 5042, South Australia; ^3^​ SA Pathology, Adelaide 5000, South Australia; ^4^​ Institute for Glycomics, Griffith University, Gold Coast, Queensland 4215, Australia; ^5^​ Infectious Disease Programme, Department of Microbiology and Immunology, Yong Loo Lin School of Medicine, and Immunology Programme, Life Sciences Institute, National University of Singapore, Singapore

**Keywords:** Dengue virus, complement factor H, complement factor B, AG129 mouse, alternative pathway

## Abstract

The complement alternative pathway (AP) is tightly regulated and changes in two important AP components, factor B (FB) and factor H (FH) are linked to severe dengue in humans. Here, a mouse model of dengue was investigated to define the changes in FB and FH and assess the utility of this model to study the role of the AP in severe dengue. Throughout the period of viremia in the AG129 IFN signalling-deficient mouse, an increase in FB and a decrease in FH was observed following dengue virus (DENV) infection, with the former only seen in a model of more severe disease associated with antibody-dependent enhancement (ADE). Terminal disease was associated with a decrease in FB and FH, with greater changes during ADE, and accompanied by increased C3 degradation consistent with complement activation. *In silico* analysis of NFκΒ, signal transducer and activator of transcription (STAT) and IFN-driven FB and FH promoter elements to reflect the likely impact of the lack of IFN-responses in AG129 mice, demonstrated that these elements differed markedly between human and mouse, notably with mouse FH lacking NFκΒ and key IFN-stimulated response elements (ISRE), and FB with many more NFκΒ and STAT-responsive elements than human FB. Thus, the AG129 mouse offers utility in demonstrating changes in FB and FH that, similar to humans, are associated with severe disease, but lack predicted important human-specific and IFN-dependent responses of FB and FH to DENV-infection that are likely to regulate the subtleties of the overall AP response during dengue disease in humans.

## Introduction

Dengue virus (DENV) is a mosquito-borne *flavivirus* for which there are four distinct serotypes, (DENV-1–4) [1, 2]. Infection with DENV can result in clinical presentations ranging from a febrile illness (dengue) to more severe disease (dengue with warning signs) and life-threatening disease with increased vascular permeability and haemorrhagic manifestations [3, 4]. Severe dengue is more likely to occur during a second infection with a heterologous serotype [5, 6], where the cross-reactive but not cross-protective immunity contributes to severe disease by mechanisms such as antibody-dependent enhancement (ADE) of infection [7]. Additionally, the immune response contributes to disease via other mechanisms. For instance, the primary pathology of dengue vascular leak syndrome is mediated by dysregulated vasoactive cytokine and chemokine responses, such as TNF-α [8, 9], and actions of the viral NS1 protein on the vascular endothelium [10, 11]. Cleavage products generated during complement activation similarly contribute to the dysregulated inflammatory response during DENV-infection [12, 13].

The complement system involves a cascade of protein cleavages that generate sequentially activated downstream proteins involved in targeting non-self components for lysis by the membrane attack complex (MAC) [14]. Additionally, complement cleavage products have direct vasoactive and chemotactic actions [15, 16]. There are three arms of the complement pathway; the classical pathway (CP), lectin pathway (LP) and alternative pathway (AP) [14]. While all three pathways can be activated by recognition of danger signals associated with infection, the AP is constitutively active but tightly regulated to prevent damage to the host [17]. There are several activators and regulators of the AP [18], with key roles played by factor B (FB), as a driver of formation of the active C3b convertase complex (C3bBb) during which process both C3 and FB are cleaved [19], and factor H (FH), a negative regulator of C3 convertase formation and activity [20]. As such, high levels of AP activity are associated with complement consumption, and low levels of complement substrates such as FB and C3 [21, 22]. Low levels or mutations in FH are also associated with overactivity of the AP and complement-mediated diseases of the vasculature and kidneys, such as atypical haemolytic uremic syndrome (a-HUS) [23] and C3 glomerulopathies [24].

DENV interacts with the complement system on a number of levels: the LP or C1q from the CP recognizes DENV virions and protects against infection [25–27], and disruption of this recognition by non-neutralizing antibody links loss of complement control with ADE and disease severity [28]. Further, DENV NS1 protein interacts with complement components, such as C1s and C4 to evade the CP and LP activation [29, 30], and with vitronectin and clusterin to inhibit terminal-pathway MAC formation [31, 32]. Interestingly, while West Nile Virus NS1 binds FH, the negative regulator of the AP, DENV NS1 does not [33, 34], suggesting that DENV does not evade the AP in the same way as other flaviviruses. Consistent with this, severe dengue is associated with increased activity of the complement AP, specifically an increase in circulating levels of the anaphylatoxins C3a and C5a [21, 22], an increase in factor D (FD) and a decrease in circulating FH [13]. Using *in vitro* models, our laboratory has shown increased activity of the AP during DENV-infection in the local environment of DENV-infected macrophages and endothelial cells, with increases in FB but not FH protein and increased C3b deposition on cells [35].

To further investigate the complement AP during DENV-infection, changes in FB and FH in a mouse model of DENV-infection were defined. Results support that in the IFN-deficient AG129 mouse model where disease severity can be experimentally modulated by ADE, FB and FH are temporally altered during DENV-infection, with the magnitude of these changes associated with disease severity. IFN, STAT and NFκΒ responsive promoter elements are important predicted regulators of FB and FH in humans and many of these promoter elements differ in mice and would not be activated in the AG129 IFN-receptor deficient mouse model. Thus, the available DENV AG129 mouse models may not reflect the overall AP-DENV interaction but still demonstrates complement responses associated with disease severity and may be useful to dissect the roles of specific factors controlling the AP during DENV-infection.

## Methods

### Clinical cohort

Human serum samples from DENV-seropositive and seronegative healthy patients form part of a retrospective descriptive study [36]. These samples were collected over a 13 month period between 1 January 2014 and 31 January 2015 and sera was obtained from archival material stored at −20 °C. Serological analysis of DENV IgM/IgG/NS1 was performed by Dengue Duo assay (SD BIOLINE) and reverse transcription (RT)-PCR and serotype identified by PCR, as described [36]. The current study includes 10 DENV-seronegative and 29 DENV-seropositive patients from the total cohort encompassing DENV-1, -2 and -3 serotypes [36].

### FH and FB ELISA

Human or mouse FH proteins were quantitated in sera using an in-house ELISA as previously described [35]. In brief, 96-well microtitre plates were coated with goat anti-human FH at 10 µg ml^−1^ or sheep anti-mouse FH antibody at 2.5 mg ml^−1^. Human and mouse serum were diluted 1 : 1000 and 1 : 500, respectively, in PBS+1 % (w/v) bovine serum albumin (BSA). A mouse anti-human FH or a rat monoclonal anti-mouse FH diluted 1 : 10 000 were used as detection antibodies for FH. Standard curves with purified human FH (10–400 ng ml^−1^) or commercial recombinant mouse FH (20–200 ng ml^−1^) were used producing a linear regression curve (R^2^ >0.99). Human FH ELISA had an intra- and inter-assay co-efficient of variation of 6.04 and 11.57 %, respectively, while mouse FH ELISA had intra- and inter-assay coefficients of variation of 4.24 and 10.98 %, respectively.

The levels of human or mouse FB were measured using the Human FB (ab137973, Abcam) or the mouse FB (SEC011Mu, Cloud-Clone) ELISA kits, respectively, in accordance with the manufacturer’s instructions.

### DENV-AG129 mouse-infection model of dengue disease

Mouse infections utilized DENV-2 strain, D2Y98P-PP1, isolated from a patient in Singapore during 2005 (GenBank accession no. JF327392) and stocks were amplified in C6/36 cells. Cell-culture supernatants containing virus were harvested, clarified, filtered (0.22 µM, Sartorius) and stored at –80 °C until use. The titre of infectious virus was determined by plaque assay using Vero cells and quantitated as p.f.u. per ml. Then, 5–6-week-old AG129 mice (129/Sv strain deficient in IFN-α/β and IFN-γ receptors) were mock-infected or infected subcutaneously with 10^4^ of the DENV-2 strain, D2Y98P-PP1. The clinical signs were scored: 1=ruffled fur, 2=hunched back, 3=severe diarrhoea, 4=lethargic, 5=moribund [37]. Serum, liver and kidney samples were harvested at day 2 and 4 post infection (p.i.) when the animals were humanely euthanized. Each group of animals consisted of *n*=6 mice except for the day 4 p.i. group where *n*=7. All tissue samples were collected directly into TRIzol reagent for RNA extraction and sera and tissue samples stored at −80 °C prior to analysis.

### DENV-AG129 mouse-infection model with ADE and severe dengue disease

Five–six-week-old AG129 mice born from previously DENV-1 (05K3903DK1, 10^6^ p.f.u., subcutaneously) infected, but convalesced mothers or DENV-naïve mothers, were either mock-infected (*n*=3 each) or infected subcutaneously with 10^3^ p.f.u. of D2Y98P-PP1 DENV-2 (*n*=5 each), as described previously [37]. In brief, the DENV-immune model involved infection of 6-week-old female AG129 mice with a non-lethal dose of a DENV-1 strain, followed by mating 1 week later and delivery of pups. Clinical signs were scored as described above. As previously reported [37], day 6 post-DENV2 infection the DENV-naïve mice had a mean disease score of 2/5 and 100% survival while mice born to DENV1-immune dams had a mean disease score of 5/5 and were ethically euthanized at this time point. Animals from the latter group contained elevated levels of circulating DENV RNA, increased vascular leakage in the intestine, liver, spleen and kidney, and increased serum levels of cytokines and chemokines including TNF-α [37]. Serum samples were collected at day 3 and day 6 p.i. Liver and kidney samples were harvested at day 6 p.i. into TRIzol reagent for RNA extraction and samples stored at −80 °C.

### RT-PCR

Total RNA was extracted from mouse tissues using TRIzol, DNase I treated and 0.5 µg RNA was reverse transcribed with 60 µM random hexamers and M-MuLV reverse transcriptase. The cDNA template was subjected to real-time qRT-PCR using iTaq SYBER green in a Rotor-gene 6000 (Corbett Research), using primers listed in Table 1. All PCRs were performed under the following conditions: one cycle of 95 °C for 5 min; 40 cycles of 95 °C for 15 s, 59 °C for 30 s, and 72 °C for 30 s; and one cycle of 72 °C for 5 min, except mouse FB PCR, which was annealed at 60 °C. All PCR reactions included high and low copy number comparative controls. Results were normalized against the reference housekeeping genes: cyclophilin or glyceraldehyde-3-phosphate dehydrogenase (GAPDH). The relative RNA level was determined by ΔCt method as described [38].

**Table 1.** Primers used for real time qRT-PCR

Target	Sequence	Accession no.	Amplicon size (bp)
**DENV-2**	F: GCAGATCTCTGATGAATAACCAAC R: TTGTCAGCTGTTGTACAGTCG	NM_AF038403.1	102
**Pan DENV**	F: AAGGACTAGAGGTTAKAGGAGACCC R: CGYTCTGTGCCTGGAWTGATG	NM_AF038403.1	102
**m-GAPDH**	F: GACGGCCGCATCTTCTTGTGC R: TGCCACTGCAAATGGCAGCC	NM_008084.3	120
**m-FH**	F: CGTGAATGTGGTGCAGATGGG R: AGAATTTCCACACATCGTGGC	NM_009888.3	248
**m-FB**	F: CTCCTCTGGAGGTGTGAGCG R: GGTCGTGGGCAGCGTATTG	NM_008198.2	264

m=mouse, F=forward; R=reverse; bp=base pairs. Y=C/T; K=G/T; W=A/T.

### SDS-PAGE and Western blot

To quantitate total protein, 2 µl of mouse sera was subjected to electrophoresis on Mini-PROTEAN TGX stain-free gels (Biorad), images collected (ChemiDOC gel imaging system, BioRad) and total protein quantitated using Image J. For analysis of C3, 2 µl of sera was subjected to SDS-PAGE (8%, 30 : 1 acrylamide: bis) and Western blot to nitrocellulose. Membranes were probed with rabbit anti-C3 antibody (Abcam Ab97462, 1/2000) and secondary goat anti-rabbit IgG-horse radish peroxidase conjugate (Pierce, 1/25000). Bound complexes were detected by chemiluminescence (BioRad, Clarity Max ECL) with signal detected (ChemiDOC gel imaging system, BioRad) and images analysed (Image J) [39].

### Histological analysis

Mouse kidneys were harvested and fixed in 10 % (v/v) buffered formalin, embedded and block mounted in paraffin. Sections were cut to 1 µM thickness, stained with haematoxylin and eosin (H and E) or Periodic-acid Schiff (PAS) stain. Sections were assessed by brightfield microscopy by an anatomical pathologist.

### Matinspector FB and FH promoter analysis

The Genomatix MatInspector software was used for *in silico* prediction of potential transcription-factor binding sites in the human and mouse FB and FH promoters. The MatInspector programme contains an in-built library comprising 634 matrices of transcription-factor binding sites with around 366 000 human, and mouse promoter sequences [40, 41]. MatInspector analysis for FB and FH was performed using sequence data for promoters associated with at least one verified coding transcript. Stringent criteria were set with a core similarity value of 0.75 and an optimized matrix similarity threshold of 0.8 (where the core sequence of a matrix, or binding site, of 1 reflects identity). To refine these initial matches down to the most probable transcription-factor binding sites, those with a matrix similarity higher than 0.85 and with at least one line of supporting evidence in Matinspector, such as experimental validation of the binding site, or a literature citation linking the transcription factor with FH or FB were considered.

### Statistical analysis

Results were expressed as the mean±sd, and statistical analyses were performed using a two-tailed unpaired Student *t*-test, one-way or two-way ANOVA or the non-parametric Kruskell–Wallis test for data that was not normally distributed or contained low values of *n*. Statistical analysis was performed using GraphPad Prism, version 8 (GraphPad, La Jolla, CA, USA). Differences were considered statistically significant if *P*<0.05.

## Results

### Changes in FB and FH are associated with dengue infection in AG129 mice

**Fig. 1.**
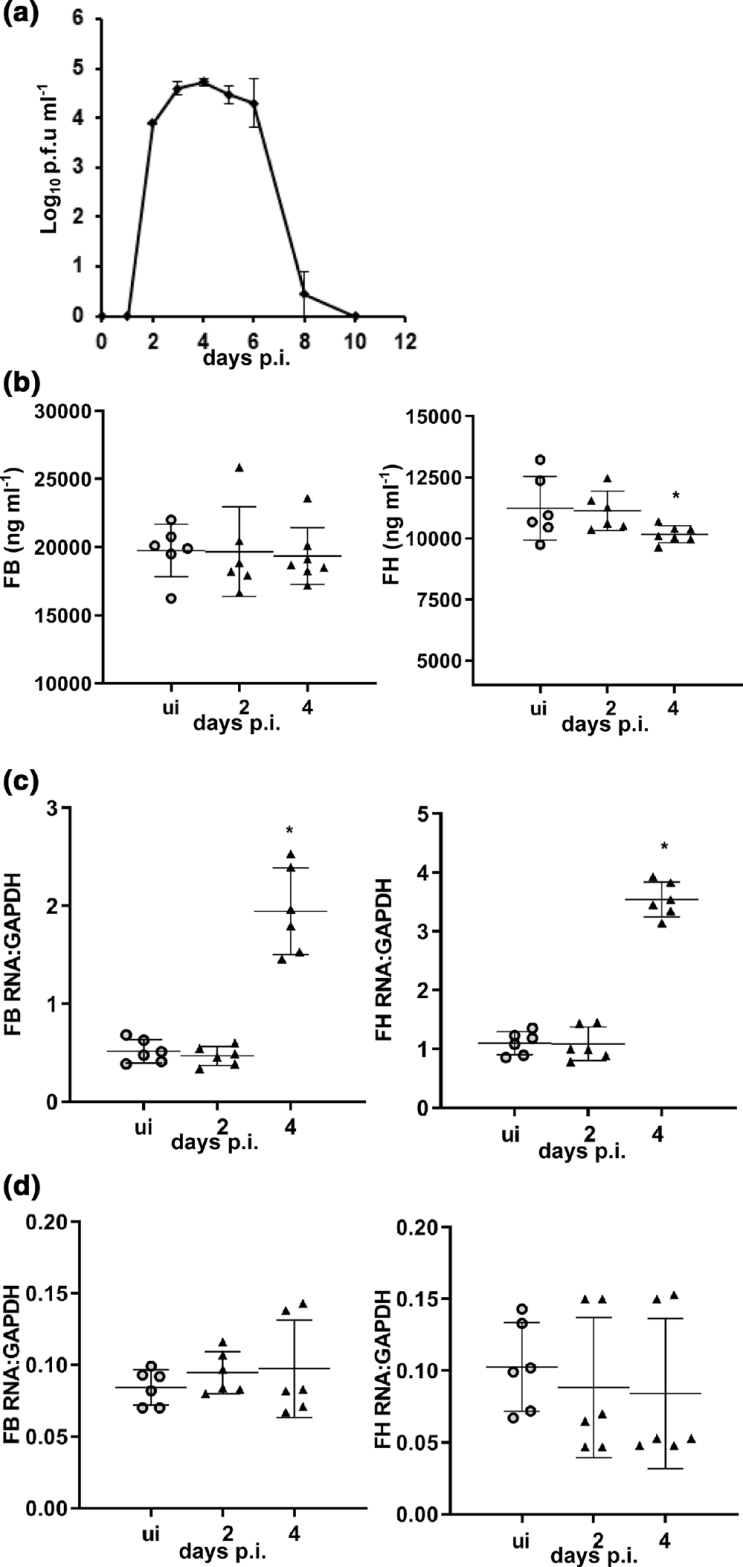
FB and FH during acute dengue infection in AG129 mice. Five- to six-week-old AG129 mice were mock-infected (*n*=6) or subcutaneously infected (*n*=6, day 2 p.i.; *n*=7, day 4 p.i.) with 10^4^ p.f.u./mouse of DENV-2 strain D2Y98P-PP1. At the indicated time points mice were euthanized, blood, liver and kidney harvested. (a) Circulating viremia was assessed by plaque assay including in *n*=4 animals kept until ethical moribund stage (day 17–21 p.i.). (b) Serum was subjected to ELISA for quantitation of FB and FH protein levels; (c) RNA was extracted from liver and (d) kidney and subjected to RT-PCR for FB and FH mRNA. mRNA levels are expressed relative to GAPDH using the ΔCt method. *P<0.05, one-way ANOVA/Tukey’s test.

To study the interaction of DENV with FB and FH in the context of severe disease a model of DENV-infection of AG129 mice, lacking IFN-α, -β and -γ receptors, was utilized in (i) an acute setting where animals succumb after the decline in viremia [42, 43] and (ii) a model of more severe disease, reflecting ADE induced by pre-existing maternal antibody and leading to a fatal disease with onset closely after the peak in viremia [37, 44]. In the model of acute DENV-infection with viremia over 2–6 days p.i. (Fig. 1a), circulating FB protein levels were unchanged while in contrast, circulating FH was slightly but significantly decreased at day 4 p.i. (Fig. 1b). This was accompanied by an increase in liver mRNA for both FB and FH at day 4 p.i. (Fig. 1c), while mRNA levels in the kidney were unchanged (Fig. 1d).

**Fig. 2.**
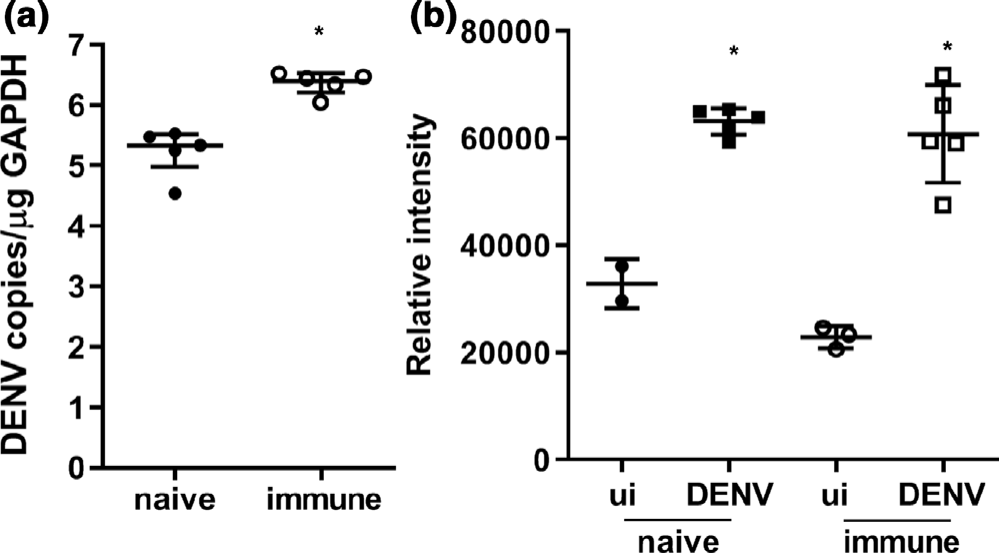
Markers of disease severity in AG129 mice infected with DENV +/-ADE. Five- to six-week-old AG129 mice born to either DENV-1-immune (immune) or dengue-naïve (naïve) mothers were uninfected (ui, *n*=3 each group) or subcutaneously infected with 10^3^ p.f.u. of DENV-2 strain D2Y98P-PP1 (DENV, *n*=5 each group). At day 6 p.i. mice were euthanized and liver and serum collected and analysed for (a) liver-associated DENV RNA with pan DENV primers by RT-PCR with normalization of DENV copy number against GAPDH. *P<0.05 Student’s *t*-test; (b) 2 µl of serum was subjected to gel electrophoresis using stain-free gels, total protein imaged by ChemiDOC and quantitated by ImageJ. Stain intensity is shown in arbitrary units (*y*-axis). *P<0.05, one-way ANOVA/Tukey’s test, relative to ui control.

In a model of dengue ADE, mice born to DENV-1 immune mothers are challenged with DENV-2 and develop a fatal disease with haemorrhage by day 6 p.i. that is more severe than DENV-2 infection in mice born to DENV-naïve mothers [37, 43]. Consistent with this, DENV liver RNA levels quantitated by RT-PCR were significantly higher in DENV-infected born to DENV-immune compared to DENV-infected born to DENV-naïve mice (Fig. 2a). Additionally, DENV-infected mice have higher circulating total protein levels (50–100 kDa range) as quantitated by gel electrophoresis of serum and stain-free imaging (Fig. 2b). This potentially reflects reduced blood volume secondary to the described vascular leak in both models, although does not reflect the greater vascular leak described with more severe disease in the DENV-immune model compared with DENV-naïve mice [37, 43].

**Fig. 3.**
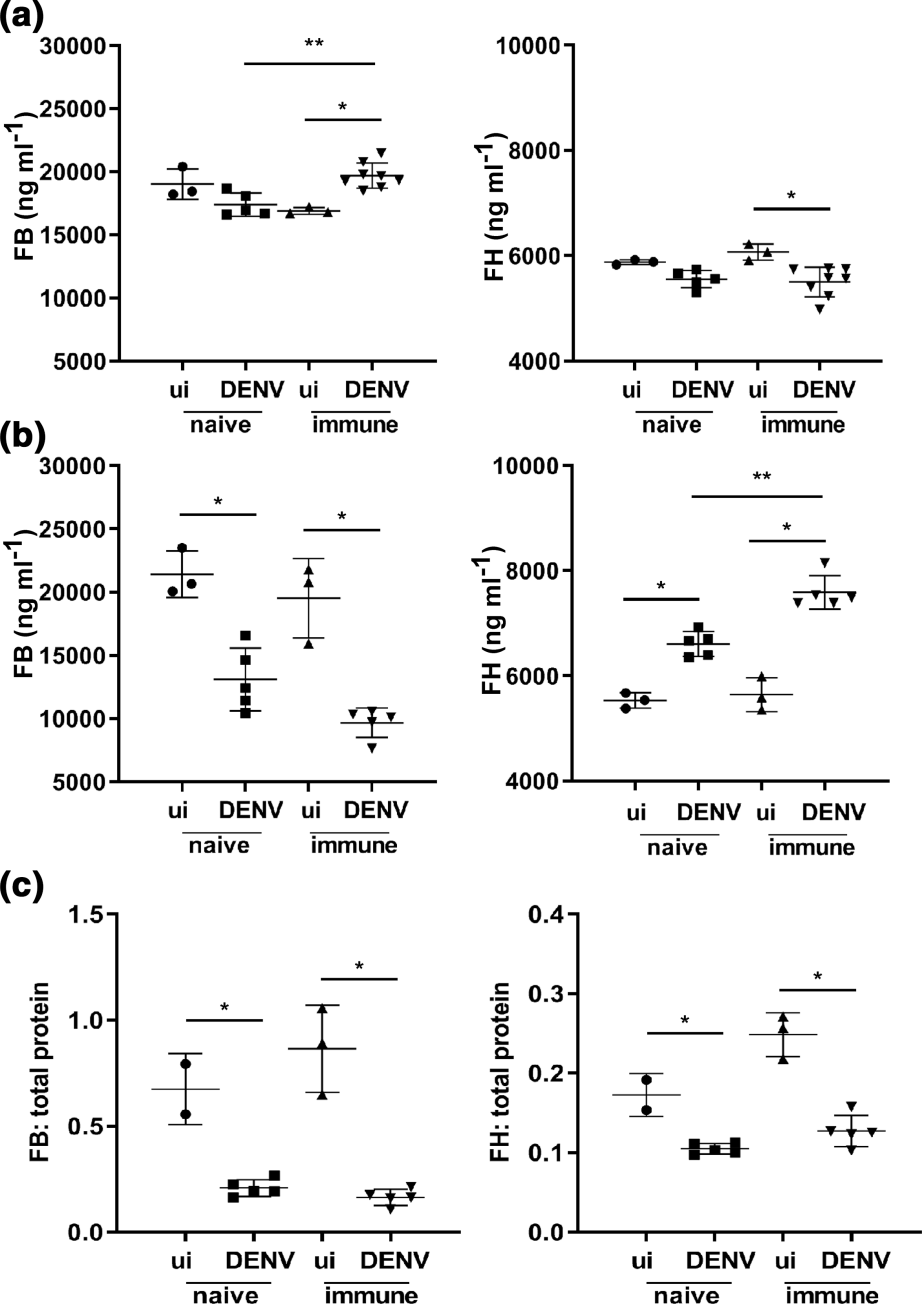
FB and FH in AG129 mice infected with DENV +/-ADE. Five- to six-week-old AG129 mice born to either DENV-1-immune (immune) or dengue-naïve (naïve) mothers were uninfected (ui, *n*=3 each group) or subcutaneously infected with 10^3^ p.f.u. of DENV-2 strain D2Y98P-PP1 (DENV, *n*=5 each group). Blood and tissue was harvested, serum collected and subjected to ELISA for quantitation of FB and FH protein levels at (a) day 3 p.i. and (b) day 6 p.i.; (c) normalization of circulating FB and FH at day 6 p.i., against total protein from Fig. 2b. *P<0.05, one-way ANOVA/Tukey’s test.

Next, circulating FB and FH levels were quantitated by ELISA at day 3 and 6 p.i. in this model. Results demonstrate a small but significant increase in FB and decrease in FH at day 3 p.i. in DENV-2-infected born to DENV-1-immune mothers (Fig. 3a). In contrast, at day 6 p.i., FB is decreased, and FH concentration increased in the circulation with a significantly greater change in FH in DENV-2-infected mice born to DENV-1-immune compared to DENV-naïve mothers (Fig. 3b). Given the increased protein concentration in blood at day 6 p.i. (Fig. 2b), results were normalized against total circulating protein, demonstrating an even greater decline in FB but negating the apparent increase and demonstrating a significant decrease in FH (Fig. 3c). No change was seen in mRNA for FB or FH in the kidney or liver at day 6 p.i. (data not shown).

**Fig. 4.**
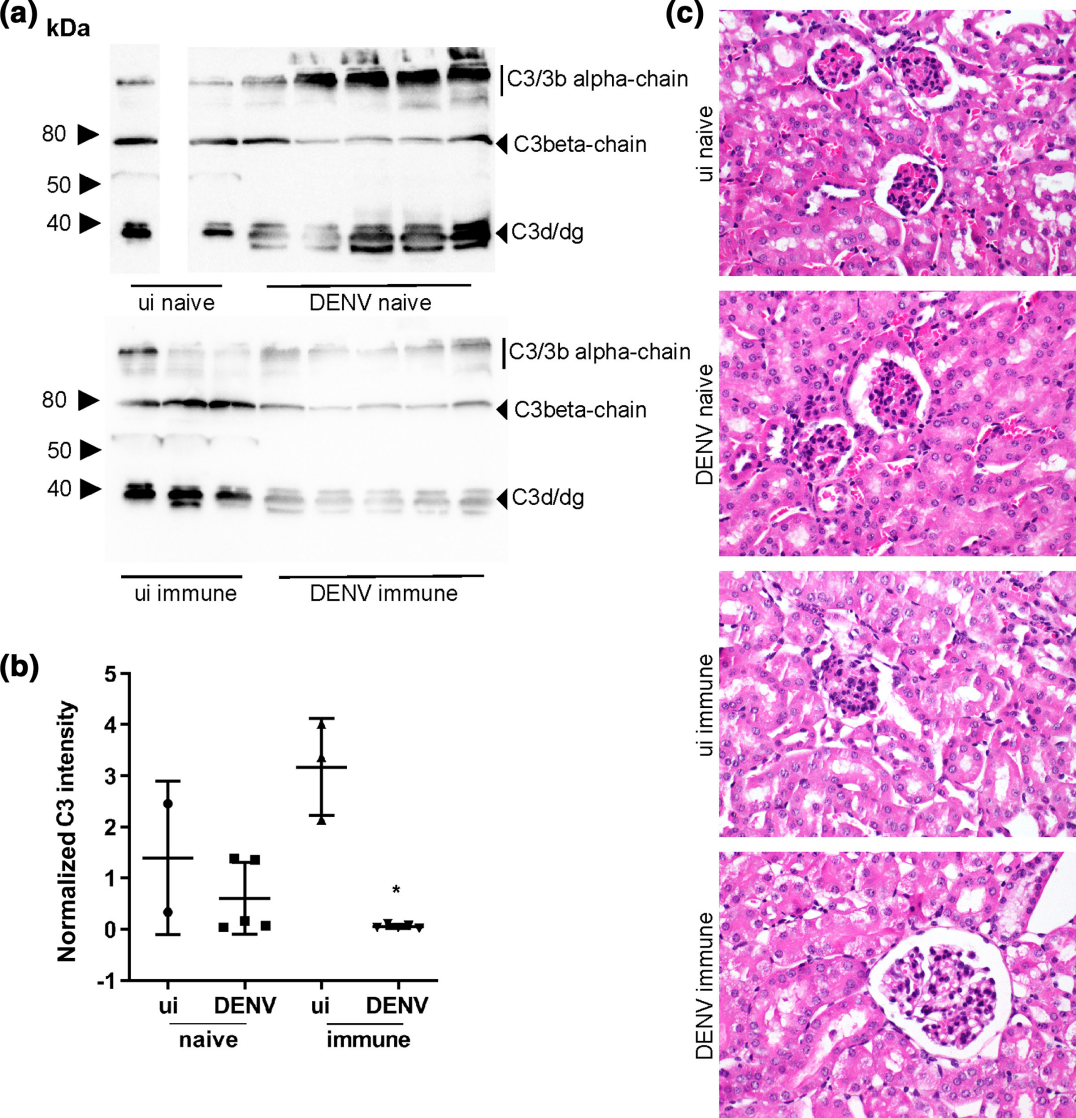
Enhanced complement activity in sera without complement-mediated pathology in the kidney in AG129 mice DENV-infected +/-ADE. (a) Serum was subjected to reducing SDS-PAGE and Western blot for C3, with detected of complexes by chemiluminescence and ChemiDOC imaging. Molecular weight markers and presumptive C3 products are indicated. ui naïve (*n*=2); DENV naïve (*n*=5), ui immune (*n*=3); DENV immune (*n*=5). (b) Results were quantitated using ImageJ and C3 β-chain normalized against total protein determined by stain-free gel imaging. *P<0.05, one-way ANOVA/Tukey’s test; (c) kidneys were fixed, embedded, sectioned and subjected to haematoxylin and eosin staining. Representative images from each group are shown, highlighting normal morphology of glomeruli and tubules.

To further define complement activation, circulating C3 was assessed by Western blot at day 6 p.i. Bands representing the potential C3/C3b α-chain (114 kDa), C3 β-chain (75 kDa) and C3 degradation productions (C3d, C3dg, 35–40 kDa) were observed (Fig. 4a). In DENV-infected animals the profile of C3 degradation products was different with increased C3d/dg in both DENV-naïve and immune mice (Fig. 4a). Quantitation of C3 β-chain or C3 d/dg (data not shown) demonstrated lower levels of C3 immunoreactivity in DENV-2-infected mice born to DENV-1-immune but not naïve mothers (Fig. 4b). Since complement-mediated pathology often manifests in the kidney, kidneys harvested at day 6 p.i. were fixed, stained and analysed by light microscopy. Representative images are shown (Fig. 4c). Kidneys showed no pathological damage, specifically no increase in glomerular obsolescence, identifiable thickening of basement membrane on PAS- or silver stains (data not shown), tram-tracking or spiking and no mesangial expansion or increase in cellularity. There was no extracapillary proliferation, no tubular damage and no proteinaceous casts identified. There was no evidence of vasculitis, granulomas, viral inclusions, papillary necrosis and no interstitial inflammation.

### Changes in factor B and factor H are not observed in clinical samples from primary dengue patients

**Fig. 5.**
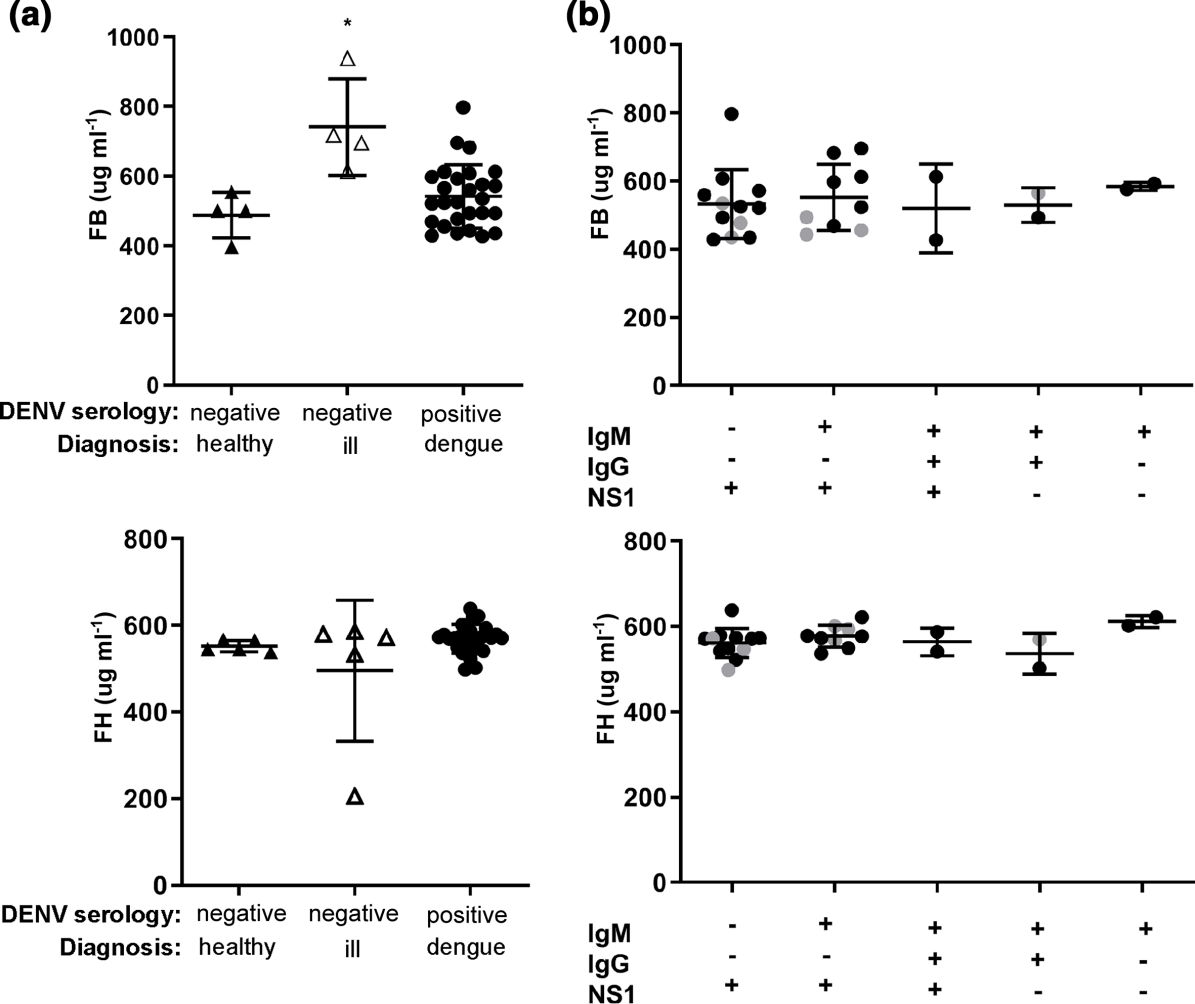
Circulating FB and FH levels in a human cohort of non-severe dengue. Samples sent for diagnostic DENV serology were analysed for FB and FH by ELISA. (a) Samples were grouped based on serology and clinical diagnosis as DENV-seronegative healthy (*n*=5), DENV-seronegative ill (*n*=5) and DENV-seropositive (*n*=29); (b) DENV-seropositive samples were stratified based on NS1, IgM and IgG status. Samples that were RT-PCR negative are indicated by grey dots. FB (upper panel) and FH (lower panel) were assessed by ELISA. Differences between groups were analysed by Kruskal–Wallis test, and are indicated by **P<0.05.

Studies in humans have shown changes in FB and FH in the context of DHF/DSS but not dengue fever [13]. Here circulating FB and FH were quantitated by ELISA in a cohort of 39 returned travellers for whom there was a clinically requested DENV serological test and reflecting primary, non-severe dengue fever [36]. Samples were grouped in accordance with DENV serology as DENV-seronegative or DENV-seropositive for any of NS1/IgM or IgG. Samples were further stratified clinically into (i) DENV-seronegative for whom no significant clinical diagnosis or laboratory findings were reported (e.g. benign headache, afebrile, C-reactive protein [CRP]<8 mg l^−1^ with normal full blood examination results), (Fig. 5a, DENV negative, healthy); (ii) DENV-seronegative who demonstrated a clinically relevant infectious diagnosis (e.g. *Salmonella enterica* infection), or laboratory result such as elevated white-cell count or CRP, (Fig. 5a, DENV negative, ill); and (iii) DENV-seropositive and clinically diagnosed with dengue fever, representing a cohort of adults with primary dengue infection and a febrile illness, as described previously (Fig. 5a, DENV positive, dengue) [36]. FB was significantly elevated in the DENV seronegative, ill group, relative to the DENV-seronegative, healthy controls or the DENV-seropositive group, (Fig. 5a). No significant changes in FH were observed in any group (Fig. 5a). Within the DENV-seropositive group, FB and FH were positively correlated (*P*=0.04, r=0.4) but neither correlated with laboratory parameters: platelet, total white cell, lymphocyte, neutrophil and monocyte counts, or CRP (data not shown). The DENV-seropositive group was stratified further based on DENV NS1/IgM/IgG serology and the presence of circulating viral RNA: 22/29 samples were DENV RT-PCR positive with the exceptions as indicated (Fig. 5b) and the majority of infections presented as RT-PCR, NS1 and/or IgM positive and thus in the early stages of infection. Most infections were DENV-1 and -2 with one DENV-3 case [36]. Although numbers in some groups were small, no significant difference or trend was observed in FB or FH levels in any DENV-seropositive group (Fig. 5b). This confirms the prior reported lack of change in circulating FB and FH during non-severe dengue, re-enforcing that the changes in FB and FH are likely specific for severe disease in humans [12, 13] and as shown here also in mice with severe dengue.

To assess potential species-specific responses of FB and FH to infection, the promoters of mouse and human FB and FH were compared with a particular focus on inflammatory and IFN-driven responses.

### Transcriptional regulation of FB and FH differs in human and mouse and is regulated by IFN

**Fig. 6.**
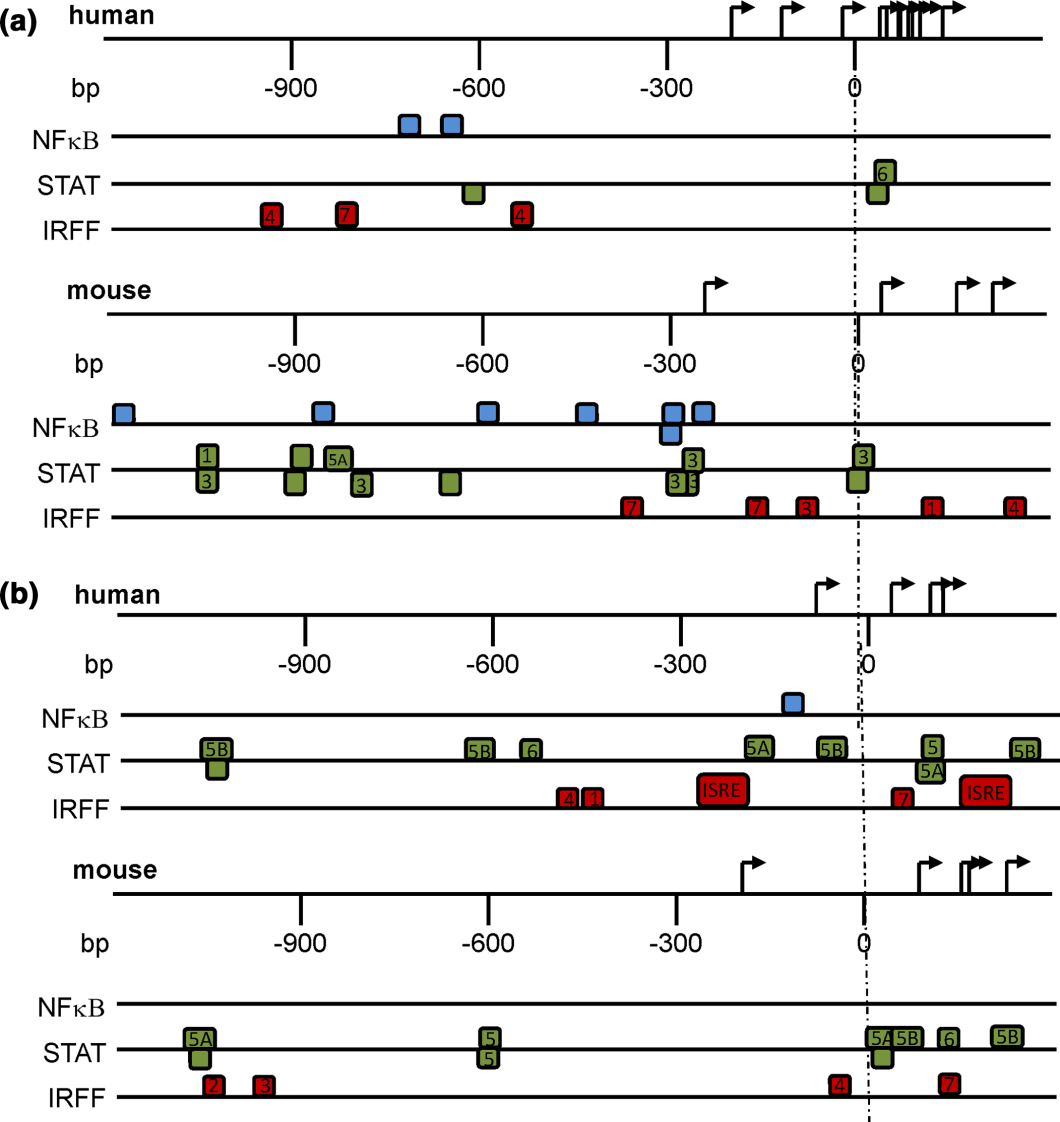
Predicted transcription-factor binding sites in the FB and FH promoters. Schematic localization of the NFκΒ, IRFF and STAT transcription-factor binding sites in the mouse and human (a) FB and (b) FH promoter sequences. Transcription-factor binding sites were identified using MatInspector software with core similarity of >0.85.

*In silico* analysis of the mouse and human FB promoter using MatInspector demonstrated NFκΒ, STAT and IRFF matrix family elements. Proximal STAT elements were present in both human and mouse promoters with overall more elements present in the mouse FB promoter (Fig. 6a). The mouse FB promoter contains five proximal IRF elements but only three, more distal elements are found in the human promoter. The mouse FB promoter contains four predicted NFκΒ sites around the −300 region and a further three >−600 while the human FB promoter contains only two NFκΒ binding sites at around −600 relative to the transcription start site (Fig. 6a). For the IRFF family, IRF4 and 7 are predicted to regulate both mouse and human FB promoters, while IRF1 and IRF3 transcription-factor binding sites are present in the mouse but not human FB promoter (Table 2). Of note, STAT3 binding sites were predicted in the mouse FB promoter, while this transcription factor was not predicted in the human FB promoter (Table 2). Overall, the mouse FB promoter contains more potential STAT and IRF transcription-factor binding sites and many more NFκΒ binding sites than the human FB promoter (Table 2).

**Table 2.** Comparison of NFκΒ, IRFF and STAT binding elements in the human and mouse FB and FH promoters

TF binding family	NFκΒ	IRFF						STAT						
Specific factor	NFκΒ	IRF1	IRF2	IRF3	IRF4	IRF7	ISRE	STAT	STAT1	STAT3	STAT5	STAT5A	STAT5B	STAT6
Human FB	2	–	–	–	2	1	–	2	–	–	–	–	–	1
Mouse FB	7	1	–	1	1	2	–	3	1	6	–	1	–	–
Human FH	1	1	–	–	1	1	2	1	–	–	1	2	3	1
Mouse FH	–	–	1	1	1	1	–	2	–	–	2	2	2	1

Predicted dependency on IRFF and STAT regulation is seen with the human and mouse FH promoter. Strikingly no NFκΒ or ISRE binding sites are predicted in the mouse FH promoter (Fig. 6b, Table 2). Several common members of the IRFF and STAT families were predicted in both the human and mouse FH promoters (Table 2), with IRF4 and 7, STAT5 and 6 predicted in the FH promoters from both species (Table 2). IRF1 was predicted in the human and IRF2 and IRF3 in the mouse FH promoter only. Overall, there is a lack of ISRE, IRF1 and NFκΒ transcription-factor binding sites in the mouse compared to the human FH promoter (Table 2). Together with the FB analysis these findings predict IFN as an important regulator of these genes and that mouse and human FB and FH are likely to respond differently to infectious challenges that stimulate IFN.

## Discussion

Complement is vital for pathogen control and the complement system is required for protection against DENV-infection. The AP, however is dysregulated and may be part of the pathology of dengue disease with lower levels of circulating FB in patients with severe DENV disease during the shock period [21, 22], and reduced FH in DHF patients compared to dengue fever patients or healthy individuals [13].

Here changes in FB and FH were defined during severe disease, to assess changes in these proteins and define the utility of an available mouse model of dengue to study the AP. Immunocompetent mice are poorly susceptible to DENV-infection and disease since the virus fails to block anti-viral signalling pathways in mouse cells [45]. AG129 mice lacking type I and II IFN receptors are permissive to DENV-infection resulting in disease with some similarities to dengue in humans [43, 46], while a model of DENV-infection in mice born to mothers with heterotypic DENV immunity induces severe dengue, as seen during secondary infection in humans and reflecting ADE [37]. In the DENV-AG129 mouse-infection model where mice survive the acute viremia, circulating FB protein levels are unchanged while FH protein was reduced at day 4 p.i. In a more severe dengue model with ADE of infection, the mice succumb to disease at day 6 p.i. concomitant with viremia, and this is associated with an early increase in FB (day 3 p.i.) while FH levels significantly decrease. Later in infection, circulating FB levels decrease (day 6 p.i.) while FH demonstrates an apparent increase in concentration that is associated with a generalized increase in circulating protein. When normalized for total protein, circulating levels of FH are actually decreased.

The changes in FB and FH are significantly greater in mice defined previously to have more severe disease when infected under ADE conditions [37, 43]. The early increase in FB and subsequent decrease at terminal disease stage in mice is consistent with activation of FB as an acute phase reactant, then subsequent decrease in FB due to complement consumption, in agreement with the decline in FB observed in DHF patients during the shock phase [21, 22]. The early decrease in FH levels in mice is consistent with findings of reduced FH in DHF cases [13]. While direct quantitation of C3 split products C3a or C3b would have also been of value here, our findings of reduced C3 and increased C3 low molecular weight fragments (C3dg), are consistent with complement activation [47] and the above described changes in FB and FH. C3 levels have previously been shown to be reduced in severe dengue [21], and are lower in patients with DHF compared to DF [13]. Thus, the AG129 dengue-mouse model appears to reflect FB, FH and C3 changes described in the literature in association with severe dengue disease in humans. Consistent with this, in our human cohort of non-severe dengue changes in circulating FB and FH were not observed.

Both the vasculature and kidneys are susceptible tissues to complement-mediated pathology. The AG129-ADE model has previously been shown to be associated with a vascular leak syndrome and our data demonstrates measures of complement activation in this same model. There was, however, no evidence of complement-mediated damage to the kidney in the DENV-infected AG129 mouse model with or without ADE. Consistent with this, renal dysfunction and damage can occur during dengue in humans but is rare.

In general, circulating changes of FB and FH proteins were not reflected by mRNA changes in the liver or kidney of AG129 mice and particularly at late stages of disease, the circulating changes appear to be mediated by post-translational consumption (FB) or haemoconcentration (FH). DENV-infection however, induces FB and FH mRNA in the liver during acute viremia. This raises the question of the importance of IFN-stimulated transcriptional responses, which are absent in the AG129 mice, in this context.

*In silico* promoter analysis demonstrated the presence of NFκΒ, IRF, STAT and ISRE elements in the FH and FB promoters, strongly suggesting regulation of these genes by type I IFN and multiple cytokines such as IFN-γ, TNF-α and IL-6, as are known to be increased during DENV-infection [48–50]. The mouse FB promoter demonstrates seven NFκΒ binding sites but none in the mouse FH promoter. In contrast, the human FB has only two NFκΒ elements with a single binding site for NFκΒ in the human FH promoter. Polymorphisms in this FH NFκΒ promoter binding site have been identified in humans that link higher expression of FH with reduced severity of disease [51], suggesting this element has functional importance and thus the lack of NFκΒ-driven FH responses in mice may inherently predispose mice to increased disease severity. Additionally, the human and mouse FB and FH promoters are potentially activated through STAT3, 5 and 6 transcription factors. STAT5 can be activated by members of the IL-2 (IL-2, IL-7, IL-15, IL-21) and IL-3 families (IL-3, IL-5) [52] and IL-2, IL-21 and IL-5 are known to be induced in patients with DF and DHF [53]. The mouse FB promoter contains six predicted STAT3 responsive elements that are not present in the human FB promoter. IL-6, a cytokine significantly elevated in DENV-infected AG129 mice [37, 43] and associated with disease in humans is an activator of STAT3 [54, 55] and thus this is a pathway unique in the mouse that may induce FB during DENV-infection. Thus, in the context of DENV-infection, NFκΒ and STAT-responsive elements are likely to be activated and be important in disease, and the human and mouse FB and FH promoters have different capacities to respond to this. This suggests that the mouse model may not be a good reflection of inflammatory drivers of FB and FH during DENV-infection.

Additionally, our promoter analysis has demonstrated various IRFF promoter elements with inherent differences in human and mouse FB and FH promoters. Specifically, IRF4 and 7 are present across human and mouse FB and FH promoters. The expression of IRF4 is restricted to T- and B-cell lineages [56–58] and thus unlikely to be important directly in DENV-infected cells. IRF7 and IRF3 are considered the master regulators of type I IFN responses, with expression of IRF7 dependent on IFN-α/β signalling [59, 60] and thus, IRF7-driven responses will be absent in the AG129 mouse model. The mouse FB and FH promoters have IRF3 binding sites, but these are not found in either of the human promoters. Further, the human FH promoter contains two ISREs, key elements in induction of ISGs which are not present in the mouse FH promoter. Similarly, IRF1 is unique to the human FH promoter while the mouse FH promoter contains a unique IRF2 binding site. Together these results suggest that the mouse promoters differ to human in likely important NFκΒ, STAT and IFN-regulated elements that are anticipated to drive FB and FH responses during DENV-infection.

Thus, the AG129 IFN-receptor deficient mouse elicits some changes in FB and FH that correlate with disease severity in a manner similar to that seen during severe DENV-infection in humans but differs in promoter elements that control IFN- and NFκΒ-driven FB and FH responses that are predicted to be important. Alternative DENV-susceptible mouse models such us IFNAR^-/-^ [61], deficient only in IFNα/β receptor, could be utilized to further study roles for specific factors, such as IFN-γ in FB and FH responses. Additionally, the AG129 model could benefit the study of IFN-independent induction of acute complement responses to differing virus inoculum for instance from mosquito compared to mammalian sources with differing glycosylation patterns and potential ability to activate complement. Human cohorts, however, with well-defined timing of infection and well characterized disease severity and physiological correlates, remain a good source of data to define the changes and likely roles of FB and FH during DENV-infection.
